# Minimally invasive surgical options for ureteropelvic junction obstruction: A significant step in the right direction

**DOI:** 10.4103/0970-1591.45533

**Published:** 2009

**Authors:** Stephanie J. Symons, Victor Palit, Chandra Shekhar Biyani, Jon J. Cartledge, Anthony J. Browning, Adrian D. Joyce

**Affiliations:** Endourology Society Fellow, Muljibhai Patel Urological Hospital, Nadiad, Gujarat, India; Specialist Urology Registrar, London, UK; 1 Huddersfield Royal Infirmary, UK; 2 Pinderfields General Hospital, Wakefield, UK; 3 St James’s University Hospital, Leeds, UK

**Keywords:** Acucise, balloon dilatation, endopyelotomy, laparoscopy, pelviureteric junction obstruction, robotic

## Abstract

Open pyeloplasty is the gold standard treatment for adult ureteropelvic junction obstruction (UPJO) with published success rates consistently over 90%. In recent years, the management of UPJO has been revolutionized by the introduction of endoscopic procedures and laparoscopic techniques. We analyzed the long-term results of endoscopic and other minimal access approaches for the treatment of UPJO.

Early results for endopyelotomy were promising but long-term results were not encouraging. Laparoscopic pyeloplasty technique is well defined and duplicates the surgical principles of conventional open pyeloplasty. With such a large variety of minimally invasive procedures for the treatment of UPJO available, the treatment choice for UPJO must be based on the success and morbidity of the procedures, the surgeon’s experience, the cost of the treatment, and the patient’s choice. We feel that with the technological advances in instrumentation coupled with a decrease in cost and improved training of urological surgeons, laparoscopic pyeloplasty may evolve to be the new “gold” standard for the treatment of UPJO.

## INTRODUCTION

Indications for surgical intervention in cases of adult ureteropelvic junction obstruction (UPJO) include symptoms related to the obstruction, progressive deterioration of ipsilateral renal function, impairment of overall renal function, infection, the development of stones or, rarely, causal hypertension.[1] Thus, intervention is aimed at preservation of renal function and symptom relief. To this end, an open reconstructive operation has historically been implemented.

Open operative intervention for UPJO provides a widely patent, dependently positioned, well-funnelled ureteropelvic junction.[2] Several reconstructive techniques have been described which can be divided simply into dismembered and non-dismembered pyeloplasty, but it is the Anderson-Hynes dismembered pyeloplasty which has gained the most universal acceptance. This is because it can be applied to all types of UPJO and provides a reliable long-term success rate in excess of 90%.[3,4]

Although open surgery has stood the test of time for successful management of UPJO, the past twenty-five years in urology have seen a major shift towards more minimally invasive techniques. Technological advances in instrumentation provided the foundation for initially endourological and, more recently, laparoscopic techniques for addressing the obstructed pelviureteric junction. However, each new technique must continue to be compared critically against its open counterpart, not only in terms of reduced patient morbidity and decreased hospital stay, but also in its ability for symptom relief and the preservation of renal function.

Through this review, we aim to provide an overview of each of the minimally invasive options for UPJO treatment and to make some comment on what’s in and what’s out. Furthermore, much has been written about laparoscopic pyeloplasty in terms of its steep learning curve and limitation to specialist centers;[5,16] but with the recent rapid spread of advanced laparoscopic techniques within the wider urological community we now ask the question: is it the laparoscopic pyeloplasty which should now be viewed as the ‘gold standard’ surgical management of UPJO?

## ENDOPYELOTOMY

First described from the Institute of Urology in London as a ‘pyelolysis’,[6] the term ‘endopyelotomy’ was coined, and the technique popularized, by Badlani and Smith in 1986.[7] The procedure involves a full thickness incision made laterally through the obstructing proximal ureter, from the ureteral lumen out to the peripelvic and periureteral fat. The importance of making the incision laterally, being the avoidance of any crossing vessels. The incision is then left to heal over a double J stent. When originally described, the incision was performed using a cold knife under vision in an antegrade fashion. More recently, the procedure has been undertaken using a hot knife or Holmium laser. Endopyelotomy can also be performed ureteroscopically in a retrograde fashion and without direct vision, under fluoroscopic control, by use of the Acucise balloon. Whichever approach is adopted, initial access across the obstructing pelviureteric junction is required for a safe endopyelotomy to be performed.

The published literature contains many series of endopyelotomy, and it appears that several factors are predictive of success: the presence of hydronephrosis, degree of ipsilateral renal function, stricture length, the presence of crossing vessels, and the period of follow-up reported. These factors have mostly been studied in patients undergoing antegrade endopyelotomy, but appear to be applicable to endopyelotomy as a whole.[8] Gupta *et al*.[9] found that patients with Grade 4 hydronephrosis had only a 54% success rate when compared to the 96% success seen in patients with a Grade 2 hydronephrosis undergoing antegrade endopyelotomy. Others have shown comparative findings.[10,11] Similarly, several authors have demonstrated that patients with poor renal function have a much worse outcome than those with good renal function. Those with less than 25% relative renal function have a success rate of only 50 to 57% compared with the 90 to 92% success seen in those with greater than 40% relative function.[9–11] In terms of stricture length, it is now accepted that any stricture of 2 cm or more is unsuitable for any endopyelotomy, since the results are unfavorable.

### Direct vision endopyelotomy: Antegrade and ureteroscopic results

The published series of antegrade endopyelotomy show success rates of 65 to 93%, with mean follow-up periods of 6 to 67 months.[8] In a recent study, Dimarco *et al*. showed that success rates continue to deteriorate in the long term with a 10-year success rate of only 41% compared to 63% at 3 years. Interestingly, they showed similar long-term deterioration in patients who had undergone either open or laparoscopic surgery for their UPJO. These patients had a success rate of 85% at three years compared with only 75% ten years following intervention.[12]

The literature reflects well on the use of antegrade endopyelotomy for patients with secondary UPJO with several series showing an increased success rate in these patients than for those with primary disease.[9,13–15]

Retrograde endopyelotomy has very similar success rates to its antegrade counterpart with rates of 65.4 to 90% success shown in the larger published series.[8] Most series show a similar success rate for secondary and primary UPJO.[13,16]

In terms of complications, both antegrade and retrograde endopyelotomy have rates of bleeding varying between 1.2 and 9%. The associated bleeding can be pronounced with the need for subsequent nephrectomy reported. Sepsis and stent related problems are also common.

Direct vision endopyelotomy, by whichever route, is now an established minimally invasive treatment for UPJO. Sim *et al*. argue that the learning curve required to master the technique for the antegrade route is short, since many urologists are already familiar with percutaneous renal access through their work in percutaneous nephrolithotomy.[16] Certainly the same argument can be made for ureteroscopic endopyelotomy as most urologists frequently perform ureteroscopic procedures in both a diagnostic and treatment capacity. The most important factor for the use of endopyelotomy is that of patient selection as suggested previously.

## THE ACUCISE ENDOPYELOTOMY

In 1993, Ralph Clayman in association with Applied Medical (Rancho Santa Margarita, CA) introduced a unique innovation: a 2.8 cm long, 150 μm wide electrosurgical cutting wire mounted on an 8-mm inflatable balloon catheter (Acucise) to incise the pelviureteric junction under fluoroscopic control. The Acucise balloon can be used for both retrograde or antegrade endopyelotomy. Patient selection is identical to that used for direct vision endopyelotomy, with patients demonstrating mild pelvicalyceal dilatation and reasonable renal function benefiting most. It is also important to have preoperative anatomical assessment of crossing vessels to avoid complications and failure. Patients with previous renal surgery, or abnormal renal or ureteric anatomy should be avoided.

Acucise endopyelotomy involves initial introduction of a semi rigid 0.035 inch guidewire into the pelvicalyceal system under fluoroscopic control. The Acucise® catheter (6F and 78 cm in length) is then advanced over the guidewire until the balloon markers straddle the stenotic segment. The cutting wire of the Acucise® catheter should be directed posterolaterally and its position confirmed by C-arm fluoroscopy. The balloon is inflated with 1.0 ml of contrast material and the position reconfirmed by fluoroscopic evidence of a waist in the balloon. The cutting wire is then activated for five seconds at 75W pure cut (exceeding 100 watt energy and coagulation setting is contraindicated) and the balloon simultaneously inflated to 2.2 ml. Balloon inflation to this volume is sufficient for satisfactory tamponade and any over-inflation will result in balloon failure. Following this, the Acucise® balloon is deflated and pulled back and a post-incision pyelogram is performed to confirm division of the UPJO. If extravasation is not confirmed, the cutting wire is reactivated for an additional 3-5 s. If waisting remains present after two attempts an alternative procedure for treating the stricture should be undertaken; a third activation of the balloon at the same site is not recommended.

The published success rate of Acucise endopyelotomy varies between 61 and 93%.[17–19] Patients with both high-grade hydronephrosis and a crossing vessel respond poorly, achieving a radiographically patent UPJO in only 39% cases.[20] Just as in direct vision endopyelotomy, the most common complication of Acucise endopyelotomy is haemorrhage, the incidence of which is low at 2-4%.[17,21] Other uncommon complications include ureteric injury, infection, ureteral spasms, scar tissue formation, flank pain and inability to void. Overall complication rates in different studies varied from 3 to 34% and include urinoma, arterial/venous bleeding, UPJ avulsion and Acucise catheter malfunction, the most significant of which appears to be vascular injury resulting in loss of kidney.[17,18,21] Walz *et al*. reported technical incidents in 6 (12%) patients intra-operatively (3 cases of balloon rupture) and 7 (14%) haemorrhagic incidents (5 polar pedicles).[21]

Advantages of the Acucise catheter are reduced morbidity, operative time and hospital stay, thus decreasing overall hospital costs. In addition to this, the Acucise device initially gained rapid acceptance by many clinicians since standard cystoscopic techniques and real-time fluoroscopy are all that is required for its use. However, much has changed in endourology over the past fourteen years and the advent, and now widespread use of the flexible ureteroscope and holmium laser mean that the future role of the Acucise balloon in the management of ureteric strictures is diminished. But the speed of the procedure, overall ease and safety especially in complex strictures mean that there might still be a place for Acucise Endopyelotomy in the long-term. We feel that it should only be reserved for patients with a very mild non-dilated pelvis.

## BALLOON DILATATION

Balloon dilatation, like Acucise endopyelotomy, is performed under fluoroscopic control. First described for dilatation of UPJO in 1982, it can also be performed via both antegrade and retrograde routes. The balloon is inflated until the waisting seen on fluoroscopy disappears. The advantage of the technique is its short learning curve and also the low risk of bleeding as no incision is involved in the procedure. However, balloon dilatation as a primary treatment for UPJO does not appear to have gained popularity; there are only a handful of reported series quoting success rates 67 to 81% using varying criteria to define success.[8] No series has been published since 2004 suggesting that the technique is not used in significant numbers.

## PERCUTANEOUS ENDOPYELOPLASTY

In 2002, Gill *et al*. published a novel technique for the management of UPJO described as a percutaneous endopyeloplasty.[22] This involves a standard longitudinal endopyelotomy incision across the obstructing pelviureteric junction, which is then precisely sutured in a horizontal Heineke-Mikulicz fashion through the solitary percutaneous tract. Thus, a Fenger-plasty type repair of the UPJ is achieved. The potential advantages of this technique over conventional antegrade endopyelotomy include: wider caliber reconstruction of the UPJ, full thickness healing with primary intent, minimal urinary extravasation and a shorter stenting period. A financial disadvantage of the technique is the requirement for a laparoscopic suturing device to complete the horizontal suture line.

Following the initial description undertaken in nine patients, intermediate one year follow-up data by the same group showed resolution of symptoms and unobstructed drainage on an IVU and/or renography in 100% of patients.[23] These results when compared with standard endopyelotomy brought the conclusion that the endopyeloplasty may have a functional superiority over the non-sutured technique. However, the group is small and no further published literature is available to date on this technique, suggesting that it has not been taken up in any numbers by the urological community as a whole. Interestingly, Gill’s group have more recently demonstrated that it is also technically feasible to perform a percutaneous Anderson-Hynes type dismembered endopyeloplasty.[24] To date this work has been published only in animal models.

## LAPAROSCOPIC PYELOPLASTY

Schuessler first described laparoscopic management of the obstructed UPJ in 1993.[25] Following this it soon became established as both a safe and efficacious technique in expert laparoscopic hands. The main advantage of a laparoscopic approach to UPJO over the minimally invasive alternatives described above is the ability to replicate each step of the open surgical procedure. Thus, laparoscopy provides a combination of the excellent success rates of open surgery with the advantages of decreased pain, short hospital stay, and an early return to full activity for the patient.

However, fourteen years ago, laparoscopic surgery within urology was in its infancy and the complexities of intracorporeal suturing techniques were the domain of few within the wider urological community. This led initially to some scepticism about the applicability of the technique within urology as a whole.[5] In addition, the short follow up times in initial published series has meant that the verdict has been out on whether laparoscopic pyeloplasty truly matches the surgical outcome of its open counterpart.

Laparoscopic pyeloplasty can be performed via both a transperitoneal and a retroperitoneal route. The preferred approach is usually dictated by the training of the surgeon involved, but many urologists find that the increased working space and the more familiar anatomy provided by the transperitoneal approach gives it a distinct advantage. Keeley and associates found that despite initial extensive training in retoperitoneal laparoscopy, the results using this approach were lower than expected, leading them to adopt the transperitoneal approach after 17 cases.[26]

In the transperitoneal approach, the ureteropelvic junction can be accessed in either a retrocolic or a transmesenteric fashion. Kavoussi and associates state that the solitary indication for transmesenteric access to the UPJ in their hands is recognition of the renal pelvis and/or ureter through a relatively transparent descending colonic mesentery.[27] In a retrospective review of cases, they found that the transmesenteric route was more commonly applied in younger individuals and males, and for pathological conditions on the left side and malrotated kidneys. The technique was found to decrease operative time by a mean of 22.5% without an increase in complications.

The pyeloplasty itself can vary from the Anderson-Hynes dismembered pyeloplasty to the Y-V plasty, Culp pyeloplasty and Fengerplasty. The indications for each are identical to that with open surgery and, just as in open surgery, the almost universal applicability of the dismembered pyeloplasty to different clinical scenarios means that it remains the most commonly performed technique. Although several devices, such as the Endostitch, fibrin glue, and laser welding have been developed to aid the technical demands of intracorporeal suturing, surgeons have, in general, mastered the technique and rely on the less expensive standard suturing materials.

Table 1 summarizes the currently available English language literature on laparoscopic pyeloplasty including only those series with 40 or more patients. The most common complications are bleeding, anastomotic leakage, and stricture formation. The conversion rate to open surgery varies from 0 to 6.4%. However, comparison of such series remains dependent on the completeness of the data reported; vigilance for perioperative complications, as well as the definition of a complication may vary between institutions and surgeons. In a review of 2,775 urological laparoscopic procedures, Permpongkosol sensibly called for standardized reporting of laparoscopic complications in urology and suggested use of a modified Clavien classification system to fulfil this aim.[28]

**Table 1:** Results of laparoscopic pyeloplasty

Series	Procedures (n)	Approach	Operating time (min)	Hospital stay (days)	Success rate (%)	Follow-up (months)	Complications	Imaging modality used to define success (n)
Romero *et al*.[27]	170	Transperitoneal	175.9 (64-345)	2.7 (2-14)	94.1	22 (2-73)	0% conversion,	Preop: NA
								Postop: renogram or IVU
Chammas *et al*.[29]	100	Robotic Trans	Gp 1 122 (60-330)		100	Gp 1 17.5 (6-36)	0% conversion, 0% complication	Gp 1: pre and post op renogram
			Gp 2 127 (80-210)			Gp 2 40.4(21-60)	2% conversion, 6% complication	Gp 2: preop IVU + CT / postop IVU
Rassweiler *et al*.[30]	143	Retroperitoneal	125 (37-368)	5 (3-10)	94.4	63 (3-137)	0.7% conversion, 6.3% complication	Preop: IVU + USS + renogram + RGP
								Postop: IVU + renogram
Moon *et al*.[31]	170	Extraperitoneal	140 (58-290)	3 (2-14)	96.2	12	0.6% conversion, 7.1% complication	Preop: renogram/retrograde pyelogram
								Postop: renogram
Davenport *et al*.[26]	66	Transperitoneal	224 (110-340)	3.6 (1-14)	92	15 (3-38)	0% conversion, 15% complication	Preop: NA
								Postop: renogram
Madhani *et al*.[32]	93	Transperitoneal	179.4 (80-350)	4 (2-7)	93.3	12 (3-27)	6.4% conversion, 18.4% complication	Preop: USS + IVU + renogram
								Postop: renogram
Lopez-Pujals *et al*.[33]	47	Transperitoneal	341.6 (200-717)	2.25 (1-3)	93.6	19.93 (2-55)	2.1% conversion, 6.4% complication	Preop: IVU + CT + renogram
								Postop: renogram
Cutting *et al*.[34]	40	Retroperitoneal	236	3.4	85		5% conversion	Preop: IVU + renogram
								Postop: IVU + renogram
Atug *et al*.[35]	37	Retroperitoneal (primary)	219.4(130-345)	1.1	100	10.7(3-20)	0% conversion	Preop: IVU or renogram/retrograde pyelogram
	7	Retroperitoneal secondary)	279.8(230-414)	1.2	100	13.5(3-29)	0%conversion	Postop: IVU andor renogram
Patel[36]	50	Retroperitoneal	122 (60-330)	1.1	96	11.7	0% conversion	Preop: renogram
								Postop: renogram
Janetschek *et al*.[37]	67	Retroperitoneal / Transperitoneal	119 (90-210)	4.1 (2-7)	98.5	25 (4-60)	1.5% conversion, 1.5% UTI,	Preop: NA
Klingler *et al*.[38]	40	Transperitoneal	NA	5.9±2.1	87.5	23.4 (6-42)	2.5% urinoma, 5% reoperation, 2.5% stricture	Preop: IVU and Renogram
	Primary-37 Secondary-3							Postop: IVU and renogram
Soulie *et al*.[39]	55	Retroperitoneal	185 (100-260)	4.5 (1-14)	87	14 (6-44)	5.4% conversion, 5.4% hematoma, 1.8% urinoma, 1.8% pyelonephritis, 3.6% anastomotic stricture	Preop: IVU
	Primary-54 Secondary-1							Postop: IVU
Turk *et al*.[40]	49 (all Primary)	Transperitoneal	165 (90-140)	3.7 (3-6)	98	23 (1-53)	2% anastomotic leakage	Preop: IVU and Renogram
								Postop: IVU and renogram and USS

IVU-intravenous urogram, USS-ultrasound

In terms of outcome, the success rates shown by these larger series vary from 85 to 100%. However, the criterion for pre-operative and post-operative assessment of UPJO has not been standardized and not all series have used renography in their assessment of surgical success. It could be argued, therefore, that individual series cannot be readily compared in terms of their results. In addition, success rates should be further subdivided for laparoscopic pyeloplasty performed for primary and for secondary pelvi-ureteric junction obstruction to assess the long-term outcomes of the technique more accurately. A further factor when comparing individual published series of laparoscopic pyeloplasty may be the patient mix in relation to adult and paediatric cases. Mandhani *et al*. presented a comparative analysis of 69 adult and 24 paediatric renal units, showing a 95.3% success rate in the adult group, but slightly lower success of 87% in the paediatric group.[32] Other series have not separated adult from paediatric cases for comparison.

In earlier reviews, one of the criticisms leveled at laparoscopic pyeloplasty was the absence of long-term follow up. It will certainly be some years before any series of laparoscopic pyeloplasty matches the mean 10-year follow-up that was demonstrated by O’Reilly and colleagues in their series of open pyeloplasties.[3] However, Jarrett *et al*.[41] show that failures following laparoscopic pyeloplasty tend to occur within the first year and Davenport *et al*.[26] showed that the mean time to failure was 4.6 months (3-11). In a large series, Dimarco recently found that the long-term success rates of both open and laparoscopic pyeloplasty, as well as endopyelotomy were not as high as has been published elsewhere. In this study, the 10-year success rate of pyeloplasty was only 75% in comparison with 85% at 3 years; which was much better than that of endopyelotomy where the 3 and 10-year success rates were only 63% and 41%, respectively. Extended follow-up reveals that a significant number of failures appear more than 3 years postoperatively. The current study illustrates that the difference in success rates of endopyelotomy and pyeloplasty continues to broaden with extended follow up. Hence, this study recommends that follow up imaging should not be abandoned. In future the minimally invasive advantage of endopyelotomy may give way to laparoscopic or robotic pyeloplasty.[12] Thus, it can now be argued that the current literature of large reviews, where the minimum mean follow-up is 11.7 months and the maximum mean is after 63 months, is certainly valid for comparison to the long-term success rates of open surgery.

## ROBOTIC ASSISTED LAPAROSCOPIC PYELOPLASTY

In view of the perceived steep learning curve involved in laparoscopic pyeloplasty, many surgeons have chosen to opt for a robotically assisted technique. Standard laparoscopy requires the surgeon to master counter-intuitive motion, and to perform complex movements with non-articulating instruments using two-dimensional visualization. Vallancien *et al*. suggested that at least 50 difficult operations, with at least one case per week during the first year, were required to master such complex laparoscopic urological procedures.[42] The introduction of robotic technology with tremor filtering, up to 1:5 motion scaling, seven degrees of freedom and true three-dimensional vision, brought a new alternative to surgeons to facilitate the transfer to laparoscopic surgery. In terms of operating time, results appear comparable with standard laparoscopy and success rates with follow-up periods matching those of non-robotic laparoscopy are now in the published domain.[29,35,36] The main disadvantage of robotically assisted laparoscopy however, is the expense, with few centers being able to stretch to the funding required.

## CONCLUSION

Using the open pyeloplasty as the gold standard, alternative methods to treat UPJO have evolved. The field of laparoscopy in urology has grown over the past 2 decades. Fifteen years after Schuessler *et al*.[25] ushered in the new era of urological laparoscopic surgery with their report of the first case of laparoscopic pyeloplasty, the procedure is now commonly offered in the western world. Laparoscopic techniques for the correction of UPJO are well defined and adhere to the surgical principles of conventional open pyeloplasty. As increasingly mature experiences are published from institutions worldwide, laparoscopic pyeloplasty is gaining acceptance and has become the standard treatment at many US and European centers. Although laparoscopic pyeloplasty yields excellent results, it is clearly a complex procedure with a long learning curve, and therefore reserved to centers with experience in laparoscopy. In contrast, retrograde endopyelotomy is less technically challenging and easier to master by the general urologist. Acucise endopyelotomy and balloon dilatation do not provide equivalent success rates to laparoscopic pyeloplasty. We feel that with the proliferation of technology coupled with a decrease in cost and improved training of urological surgeons, laparoscopic pyeloplasty may evolve to be the new “gold” standard for the treatment of UPJ obstruction worldwide.

It is important to achieve high success rates when treating UPJO. The most effective treatments are often associated with the greatest risk of complications. Thus, the importance of a patient-based decision cannot be overestimated and patient preference is always an important factor in treatment choice.
